# Drug delivery of amoxicillin molecule as a suggested treatment for covid-19 implementing functionalized mesoporous SBA-15 with aminopropyl groups

**DOI:** 10.1080/10717544.2021.1914778

**Published:** 2021-04-30

**Authors:** Haneen F. Alazzawi, Issam K. Salih, Talib M. Albayati

**Affiliations:** a Department of Chemical Engineering, University of Technology, Baghdad, Iraq; b Department of Chemical and Petroleum Industries Engineering, Al-Mustaqbal University College, Babylon, Iraq

**Keywords:** **L** COVID-19, SBA-15, amoxicillin, drug delivery, loading and release of drug, surface functionalization

## Abstract

SARS-CoV-2 is a novel coronavirus that was isolated and identified for the first time in Wuhan, China in 2019. Nowadays, it is a worldwide danger and the WHO named it a pandemic. In this investigation, a functionalization post-synthesis method was used to assess the ability of an adapted SBA-15 surface as a sorbent to load the drug from an aqueous medium. Different characterization approaches were used to determine the characterization of the substance before and after functionalization such as X-ray diffraction (XRD), Fourier transform infrared (FTIR) spectroscopy, scanning electron microscopy (SEM), nitrogen adsorption–desorption porosimetry (Brunauer–Emmett–Teller) BET surface area analysis, and thermal gravimetric analysis (TGA). Batch adsorption testing was carried out in a single adsorption device to find the impact of multiple variables on the drug amoxicillin charge output. The following parameters were studied: 0–72 hr. contact time, 20–120 mg/l initial concentration, and 20–250 mg of NH_2_-SBA-15 dose. The outcomes from such experiments revealed the strong influence and behavior of the amino-functional group to increase the drug's load. Drug delivery outcomes studies found that amoxicillin loading was directly related to NH_2_-SBA-15 contact time and dose, but indirectly related to primary concentration. It was observed that 80% of amoxicillin was loaded while the best release test results were 1 hour and 51%.

## 1. Introduction

The Drug delivery systems (DDSs) are an important science which deals with delivering medical substances to specific parts of the body. This science has developed and new technologies of delivering treatment have been discovered. Since covid-19 invaded the world, finding treatment and drug delivery techniques for the disease has become essential. DDS is a transporter that would regulate the distribution rate of drugs and target different parts of the body by adjusting components to particular groups. The process whereby a drug is supplied might just have a major effect on its therapeutic efficacy. Since the drug typically loses effect with a conventional treatment before it hits the target location within the body. DDSs are intended to keep useful benefits during the dosing interval until the required site is approached (Albayati and Jassam, 2019). The mesoporous materials are considered directly proportional to the quality and release rate of the product loaded. These substances have received great interest because of their surface chemical composition, their chemical and thermal stability, uniform structure, and adjustable pore sizes. The high measurable surface area and pore volumes, modifiable surfaces, Non-toxicity, and strong biocompatibility, and mesoporous particle morphology are affecting the release and loading on properties of the drug (Nosrati et al., 2018; Salehiabar et al., 2018; Albayati et al., 2019; Hosseini-Ashtiani et al., 2021). Choosing the correct functionalizing agent will build a basic, acidic, and hydrophobic surface of the inner channel (Alkafajy and Albayati, 2020). Besides, the functionalized surface groups may come into contact with the desirable drug via ionic interactions such as amine-modified mesoporous materials were used as a carrier for drug processing with acidic nature (Ramezani and Zare-Dorabei, 2019). Conversely, modification of the SBA-15 (Santa Barbara Amorphous) surface with acids (i.e. carboxylic groups) has increased the loading properties of drugs with basic properties (Das et al., 2012).

SBA-15 is an essential mesoporous substance with a highly ordered hexagonal topology large, regulated pores, and simple to change and unload massive molecules (Zhu et al., 2007; Albayati et al., 2016). Silanol group's presence on the canal walls gives the pure SBA-15 a weak intermolecular hydrogen bond with drugs that are not strong enough to hold and release drugs easily. The functional groups on the surface of the SBA-15 are thus important for controlling the delivery of drugs (Sasidharan et al., 2013). Another study by Doadrio et al. (Doadrio et al., 2004) have investigated SBA-15 as a drug delivery method for gentamicin. Also, Song et al. (Song et al., 2005) identified as drug matrixes SBA-15 functionalized with groups of amine. (IBU) Ibuprofen and (BSA) bovine serum albumin were selected as standard medication and loaded onto the non – modified SBA-15. The antibiotic amoxicillin was further developed by Vallet- Regi et al. (Vallet-Regı et al., 2004) with a calcined SBA-15 content. The amount of drug integrated into SBA-15 is carefully directly proportional to pH, the concentration of the solvent, and amoxicillin. The release of amoxicillin is reliant upon the material's physical state.

**Figure 1.**
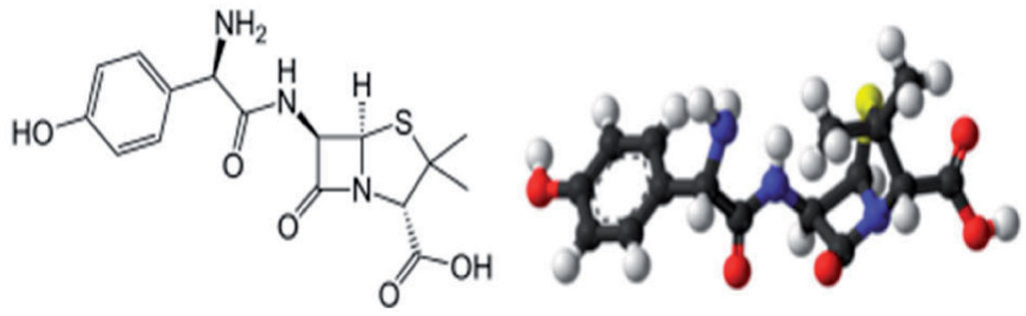
The two and three dimensional structure of amoxicillin.

According to the National Institute for Health and Care Excellence (NICE), to cover typical and multiple pathogens in older patients with pneumonia and at risk of severe complications, Amoxicillin was one of the recommended choices of antibiotics in the community. Benarousi et al (Benarousi et al., 2020) discovered Amoxicillin as very strong inhibitors with a rate of 100% to the nCov-19 main protease by several hydrogen bonds and hydrophobic interactions. Figure 1 represented the amoxicillin structure in two and three dimensions (Liu et al., 2009).

This work will discuss what drug delivery is, what coronavirus is, and how to use drug delivery techniques in developing a coronavirus treatment. This study uses functional SBA-15 to specify the loading and release of amoxicillin. The impacts on amoxicillin loading of parameters: Amoxicillin concentrations, the dosage of SBA-15, and contact time were evaluated. Amoxicillin is among the hydrophilic penicillins that are just like antibiotics. This is most frequently used in respiratory system diagnosis, like bronchitis and strep throat. This is often used for pulmonary treating bone and ear and skin infections. It is also used until surgery and by dentists to avoid possible infections, but rather to treat other bacterial infections such as pneumonia.

## 2. Materials and methods

### 2.1. Chemicals

Tetraethyl orthosilicate TEOS, (C_2_H_5_O)_4_Si, with MW = 208.33 g/mol,), Pluronic P123, (C_3_H_6_OC_2_H_4_O) with Mwt equal 5800 mol^−1^, Amoxicillin (C_16_H_19_N_3_O_5_S), Hydrochloric acid (HCl), Toluene C_6_H_5_CH_3_ with Mwt equal to 92.14 Chemicals used as part of SBA-15 synthesis work, 35% alkoxysilane [(3-aminopropyl) Triethoxy silane (APTES, C_9_H_23_NO_3_Si), also used KH_2_PO_4_=136.0855 gmol^−1^ and K_2_HPO_4_ with Mwt = 174.1759 gmo1^−1^ for PBS preparation (Phosphate Support Arrangement). Sigma Aldrich had obtained all of the ingredients. The chemicals have been used without further purification because they were provided.

### 2.2. Synthesis of SBA-15 particles

**Figure 2.**
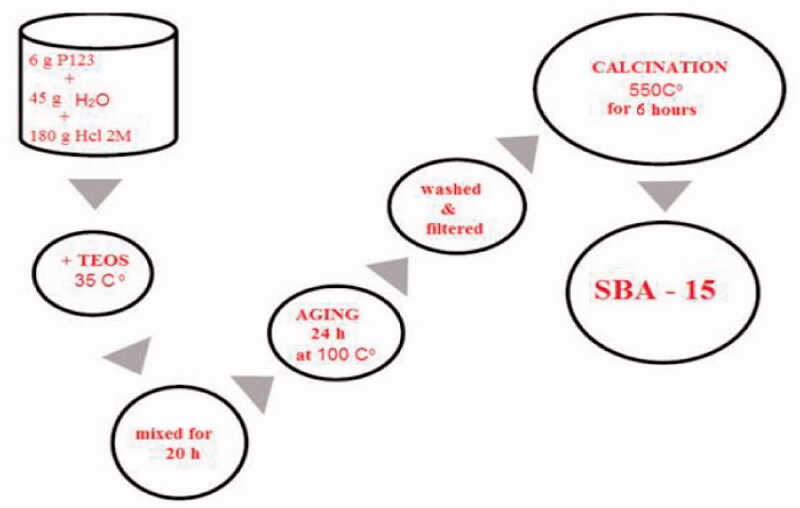
Synthesis scheme of SBA-15.

The production of pure mesoporous SBA-15 as a transport for the controlled drug delivery and as per the conventional approach was reached (Zhao et al., 1998; Albayati and Doyle, 2014) as shown in Figure 2, six g of surfactant form P123 was distributed in 45 g of distilled water. At 35 °C, in the previous solution, 180 g of 2 M HCL was added until the surfactant was fully dissolved. The gradual addition of 12.75 g TEOS to the surfactant solution was then heated at 35 °C for 20 h. The instigated concentrate was therefore stored under stable loading in a closed glass bottle for 24 hours at 100 °C. The white precipitate procured was cooled to 25^0^ C, filtered, rinsed with distilled water, and dried at 250 °C for 12 hr. SBA-15 precipitate was collected when calculations removed the surfactant in 550 °C for 6 hr.

### 2.3. Surface functionality

**Figure 3.**
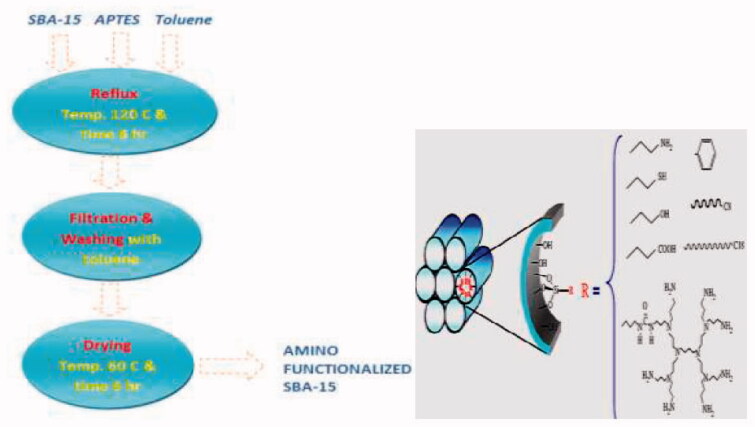
Preparation procedures of SBA-15 and NH_2_-SBA-15.

The amino-functionalized SBA-15 was developed using the procedure described by Talib et al. (Albayati et al., 2014; Albayati and Doyle, 2015; Sabri et al., 2015) using a post-synthesis process. Initially, 1 gm of calcined SBA-15 was dried for 3 hrs. At 100 °C, after that stirring with 10 ml 3-aminopropyltriethoxysilane (APTES) and 40 ml Toluene for around 6 hrs under reflux. The solution was then cooled, filtered, washed, and toluene-dried at 60 °C. A white powder of amino-SBA-15 was obtained, as shown in Figure 3.

### 2.4. Characterization

The X-ray diffract gram has been used to detect the crystal structure, identify crystalline phases, direction, and decide the physical parameters of the natural and functional SBA-15 with scanning rate 2 (deg/min) between 0° and 10°. The source of radiation from the X-ray was Cu Kα (*μ* = 1 541 Å). XRD test was managed by an X-Ray diffractometer (XRD-6000 Shimadzu) at the central service laboratory at the University of Baghdad, College of Pure Sciences Education/Ibn Al Haitham. FT-instrument is used to analyze the chemical bonds and the functional groups grafted onto the SBA-15. This analysis was achieved by the equipment type (Bruker –Tensor 27/Germany) in the Chemical Engineering Department at the University of Technology. The SEM is an important technique for the study after and before functionalization of the prepared SBA-15's structure and morphology using Electron Scanning Microscope (SEM) (Type: AIS2300C, South Korea) at College of Education for Pure Sciences/Ibn Al-Haitham at the Central Service Laboratory at the University of Baghdad. The specific BET surface area and the total pore volume for SBA-15 isotherms were collected before and after functionalization use (Brunauer, Emmett and Teller method) of nitrogen adsorption on surface area analyzer (type: Qsurf 9600, USA). Thermos Gravimetric Analysis (TGA) is a thermal conductivity analytical technique used to find functional group integration for functional SBA-15 and to obtain thermal stability information. The mixture was heated at a rate of 10 °C per min from 600 to 650 °C and analyzed using the TG-DSC thermo-gravimetric analyzer (Type: STA PT1000, Origin: USA) at the central service Laboratory in the University of Baghdad, College of Education for Pure Sciences/Ibn Al-Haitham.

### 2.5. Preparation of a solution to load amoxicillin

The drug mixture was taken as seen in Figure 1, with 1 g of amoxicillin dissolved in 1000 mL of deionized water stored in a beaker, then mix for 2 hr to create displaced amoxicillin molecules in deionized water.

### 2.6. Loading amoxicillin

The amoxicillin solution had made using 200 mg/l amoxicillin. Then, SBA-15 60 mg was combined with a solution and stirred for 72 hr. A UV analyzer was used to test amoxicillin charging at ambient temperature. The concentration change was measured at 4, 8, 12, 24, 48, and 72 hr.

SBA15 was then mixed in solution with 60 mg and stirred for 12 hr. The difference in concentration was calculated from 10 to 120 mg^−1^. 20 mg^−1^ of amoxicillin concentrations were then combined with SBA15 for 12 hr. at room temperature, the increase in concentration was measured from 20 to 250 mg at various dosages of SBA-15. The amount of amoxicillin charged was measured using the absorbance values at 272 nm (Vathyam et al., 2011; Lang et al., 2012; Bahrami et al., 2014). The percentage of the charge was determined according to the following equation:

(1) $$\text{Loading}\%=\frac{\;\mathrm{loaded}\;\mathrm{amoxicillin}\;\mathrm{drug}\;\mathrm{Weight}\;in\;NH2-\mathrm{SBA}-15}{\;NH2-\mathrm{SBA}-15\;\mathrm{loaded}\;\mathrm{Weight}\;\mathrm{with}\;\mathrm{amoxicillin}}\times 100$$

### 2.7. Release of amoxicillin

The release of amoxicillin was agreed upon using UV analysis. The test can be explained as below. Initially, they used two separate phosphate salts for the (Phosphate Buffer Solution) PBS: KH_2_PO_4_ and K_2_HPO_4_. The pH of the final volume was set to 7.4 to approximate the pH of the digestive tract (Hwang et al., 2010), then combined with 487,085 mg^−1^ amoxicillin at ambient temperature 200 mg of SBA-15. The sum of the release of the drug from the SBA-15 particles was calculated within 6 hours by Equation 2) as per the drug's shift in concentration.

% Release

(2) $$=\frac{\;\mathrm{drug}\;in\;NH2-\mathrm{SBA}-15\;\mathrm{Weight}\;}{\;\mathrm{loaded}\;\mathrm{drug}\;in\;NH2-\mathrm{SBA}-15\;\mathrm{Weight}\;}\times 100\;$$

## 3. Results and discussion

### 3.1. Characterizations of SBA-15 and NH_2_-SBA-15

#### 3.1.1. XRD analysis of NH_2_-SBA-15 and SBA-15

**Figure 4.**
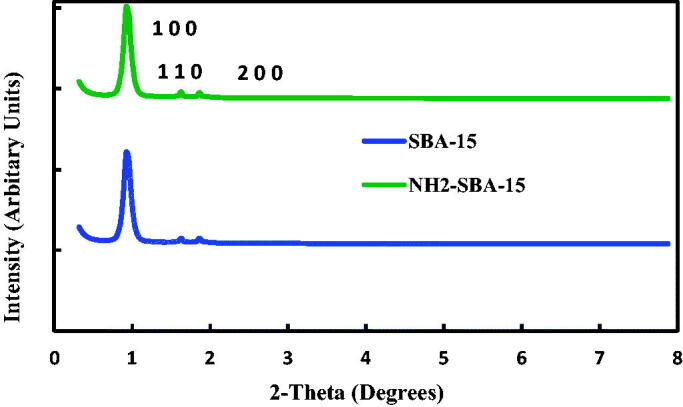
XRD patterns for SBA-15 and NH2-SBA-15.

Figure 4 displays the XRD trends for both SBA-15 and NH_2_–SBA-15 where each mesoporous material has a single peak of the high intensity (100) at 20.96°, linked to two adding different sharp peaks to (110) and (200) peaks at 2 below 2 °C, supporting the formation of a hexagonal p6 mm symmetry lattice. Therefore, inside SBA-15 mesoporous channels are grafted. XRD peak intensities of NH_2_–SBA-15 was essentially lower than those of SBA-15, likely due to the pores filling effect of the SBA-15 channels or the anchoring ligands on the SBA-15 outer surface (Albayati and Doyle, 2014).

The results demonstrate that the periodic ordered structure of SBA-15 was maintained after modification with amine group (NH_2_). However, spacing values (ɑ_o_) of the grafted SBA-15 samples reduced somewhat (Table 1), compared to SBA-15, indicating changes in their wall thickness and pore size due to the deposition of functional group NH_2_.

**Table 1.** Physicochemical properties of SBA-15 materials and NH_2_-SBA-15.

Sample	S_BET_ (m^2^/g)	V_P_ (cm^3^/g)	V_μP_ (cm^3^/g)	D_P_ (nm)	ɑ_o_ (nm)	t_wall_ (nm)
SBA-15	675	0.7908	0.06	6.16	7.29	4.13
NH_2_-SBA-15	169	0.028	0.03	7.37	5.23	3.15

#### 3.1.2. FT-IR analysis of NH_2_-SBA-15 and SBA-15

**Figure 5.**
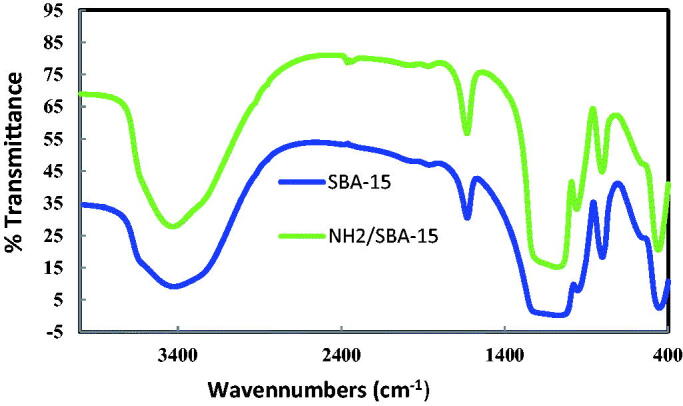
FTIR for SBA-15 & NH_2_-SBA-15.

The spectroscopy of pure SBA-15 and NH2-SBA-15 with Fourier Transforms Infrared (FTIR) can be seen in Figure 5 which deals with the friction of organic molecules in a molecule at different wavelengths, probably depends on the elements and bond types. The energy corresponding to these transitions corresponds to the infrared region (4000–400 cm^−1^) of the electromagnetic spectrum.

The adsorption into IR bands is related to the expanding vibrational mode detected on the surface in the range 3740–3500 cm^−1^ of the quiet classes. Additionally, Normally the NH extending bands are 3380–3310 cm^−1^. Consequently, these cannot be separated between absorption bands, so it appears critical to alternate NH_2_ bending when recognizing yet if or not an amine group is current. Thereby, the peak of approximately 1600 cm^−1^ divided into two other peaks could be connected to the inverted bending of NH_2_ proving an amine group as forecast. The wider band at around 1100 cm^−1^ is due to the Si–O–Si asymmetric bending vibrations instead of the symmetric stretching mode while the peak at about 800 cm^−1^ is present. Additionally, the purely and surface modification materials have small peaks to about 400 cm^−1^ which connected to the Si–O–Si matrix composite. As well, the band could have been assigned at about 950 cm^−1^ as the Si–OH bending (Alardhi et al., 2020).

#### 3.1.3. SEM analysis of NH_2_-SBA-15 and SBA-15

**Figure 6.**
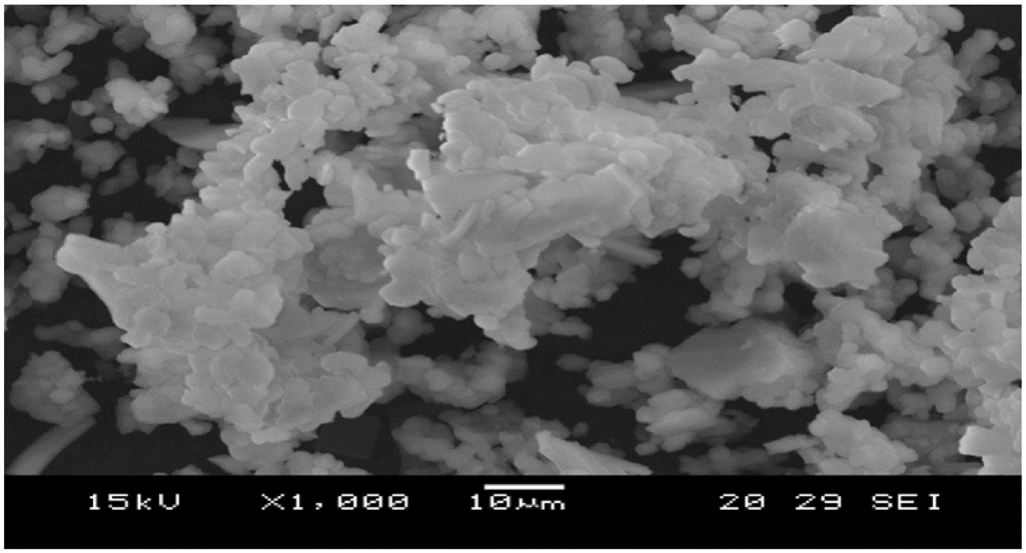
SEM images for pure SBA-15.

**Figure 7.**
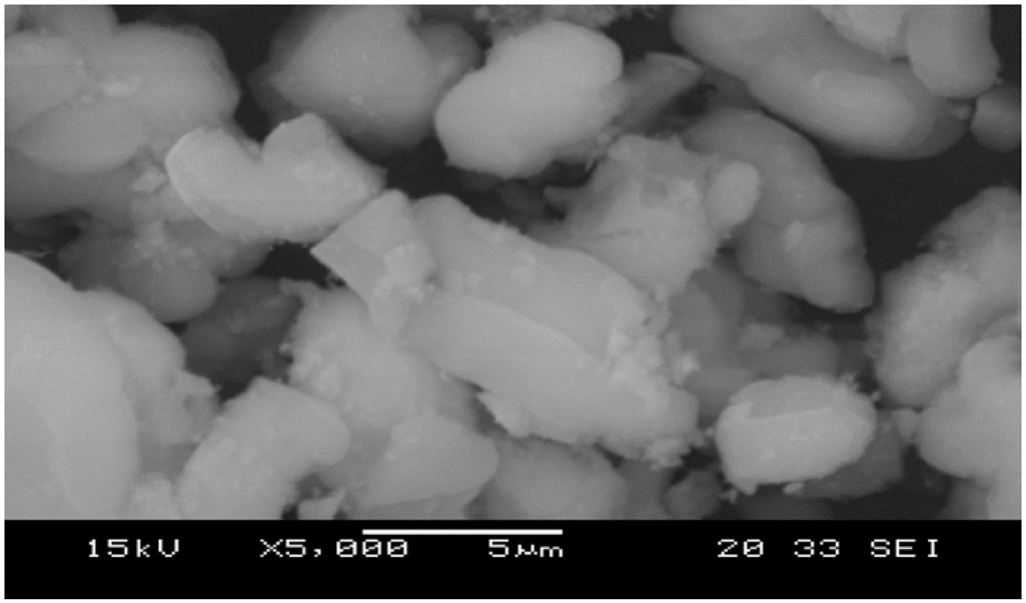
SEM images for NH_2_-SBA-15.

SEM images of raw SBA-15, amine grafted SBA-15, and morphologies are shown in Figures 6 and 7, and according to SEM images; the pore networks, rods similar crystals of SBA-15 samples were recorded well after terms and conditions, the specimens maintained their physical parameters.

**Figure 8.**
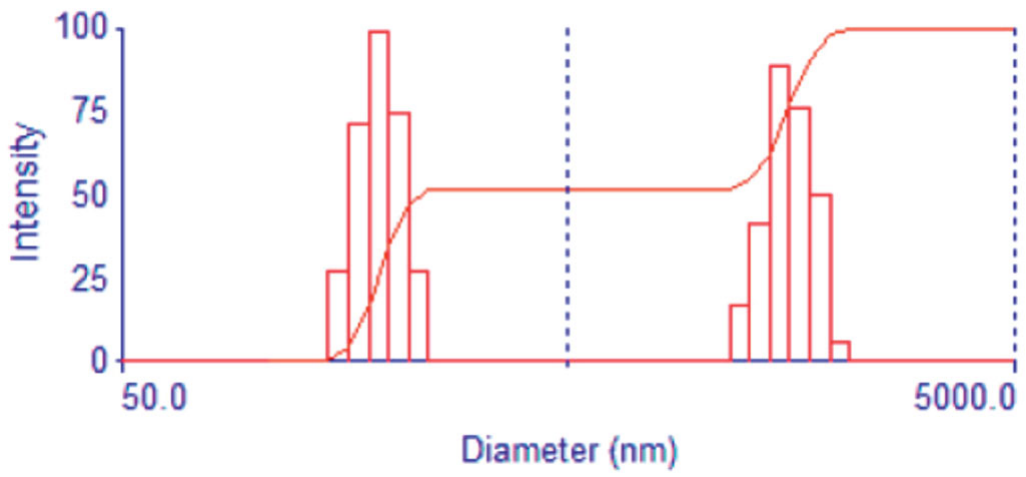
Histogram of the SBA-15 particle size distribution.

The SEM image of SBA-15 clearly illustrates the well-ordered hexagonal array construction. Closer scoping on the surface of the SBA-15 exhibited the presence of the mesoporous uniform size channels with a sphere shape puffy or swollen structures and smooth surfaces which are a typical feature of mesoporous materials (Albayati et al., 2019) The result was in agreement with the finding outline by (Albayati, 2019). These swollen structures are suitable for the absorption of the contaminated dye. A narrow pore configuration can be also recognized from the micrographs. Furthermore, Figure 8 depicts the SBA-15 particle size histograms of sample illustrating that the particles range in size from 50 to 50000 nm for sample prepared with mean diameter *845.4 nm*. The particle size determined by particle size analyzer investigation was in a good agreement with that estimated by SEM image.

#### 3.1.4. Analysis of surface area BET

**Figure 9.**
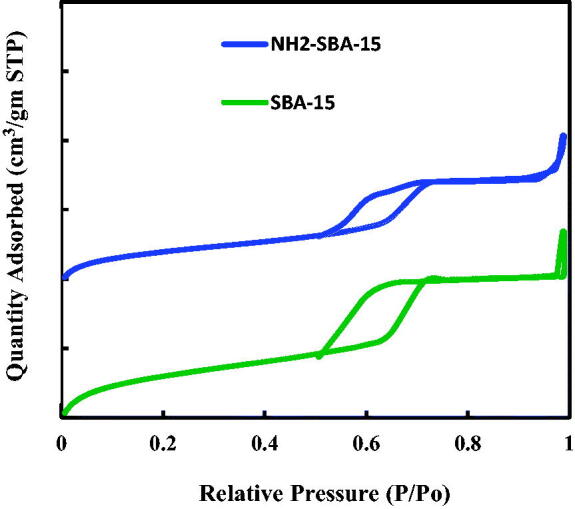
Nitrogen adsorption isotherms for SBA-15 and NH_2_-SBA-15 samples.

The nitrogen adsorption isotherms of SBA-15 and grafted NH_2_-SBA-15 materials had similar patterns as a type IV isotherm and a hysteresis loop type H1 (Figure 9); hysteresis loops with sharp adsorption and desorption branches are indicative of a narrow pore size distribution. Figure 9 also shows that the nitrogen adsorbed amount decreases as SBA-15 is grafted with NH_2_. The structural parameters calculated from nitrogen adsorption measurements are presented in Table 1. In the table, it is shown that the specific surface area, pore volume, and pore size of the samples followed the order: SBA-15 > NH2-SBA-15 whereas the different order was observed in terms of wall thickness. The significant decreases in the surface area of the loaded samples in comparison with SBA-15 confirm the attaching of functional groups NH_2_ inside the pores (Albayati and Doyle, 2014; Albayati et al., 2019; Alardhi et al., 2020). Table 1 displays the characteristics of SBA-15 and NH_2_–SBA-15. The hydrolyzing SBA-15 material was used to have a 675 m^2^g^−1^ BET surface, 0.7908 cm^3^g^−1^ pore volume, and 169 m^2^g^−1^ BET, 0.028 cm^3^g^−1^ pore volume to access SBA-15 material. Such criteria are consistent with those set out in the literature. SBA-15samples exhibited better BET surface area of hexagonal streams. The formation of binding sites on the mesoporous surface facilitates drug adsorption. On functionalization, the outcomes contribute to a decrease in the surface area and the volume of the pore. It indicates the functional population is found not only on the outer surface but also inside the mesoporous pores (Albayati, 2019).

**Figure 10.**
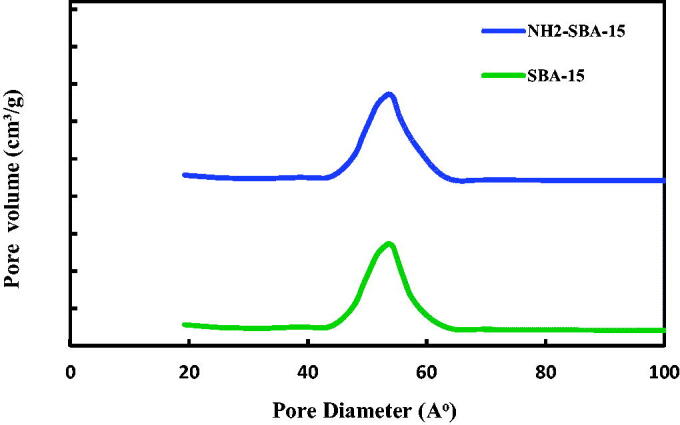
BJH PSD for SBA-15 and NH_2_-SBA-15.

Figure 10 illustrates the PSD of SBA-15 and NH_2_–SBA-15 samples. The pore size of pure silica SBA-15 is 7.29 nm, obtained from N2 physisorption using the BJH method, while the pore size of NH_2_–SBA-15 is 5.23 nm (Table 1). The NH_2_–SBA-15 shows a narrow PSD similar to SBA-15, indicating that the pore structure has not significantly altered. The decrease of the pore diameter after metals grafting is mostly resulted from the reconstruction due to amine group. This is evident in Table 1. The two sample SBA-15 and NH_2_–SBA-15 shows a PSD, with the single peak centered at around 58 Å (Albayati and Doyle, 2014).

#### 3.1.5. TGA analysis of SBA-15 and NH_2_-SBA-15

**Figure 11.**
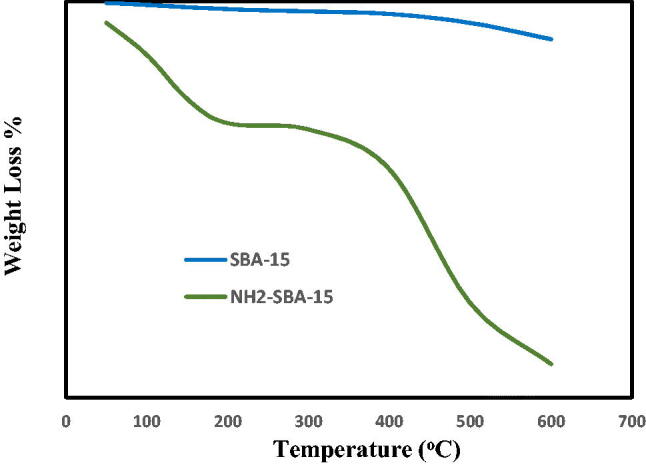
Thermal gravimetric analysis for SBA-15 and NH2-SBA-15.

The weight loss of the thermal gravimetric analysis can be seen in Figure 11 of SBA-15 and NH_2_-SBA-15 were heating from ambient temperature to 650 °C at a value of 10 °C/min. 643 °C the mass losses for pure SBA-15 were 2.919% that can be viewed as a neglected value due to the extremely low mass-loss rate at this temperature. This can be explained due to the calcined SBA-15 indicating selective dismantling of the surfactant mainly during the calcination process and also SBA-15 thermal stability can be seen. The test was conducted at a lower temperature than this one took to achieve the silica melting temperature and for NH_2_-SBA-15 this was 17,635% which can also be represented due to the removal of organic moiety due to the loading of the amino-functional group on the SBA-15 sheet (Albayati, 2019).

### 3.2. Loading of amoxicillin

#### 3.2.1. The effect of time contact

**Figure 12.**
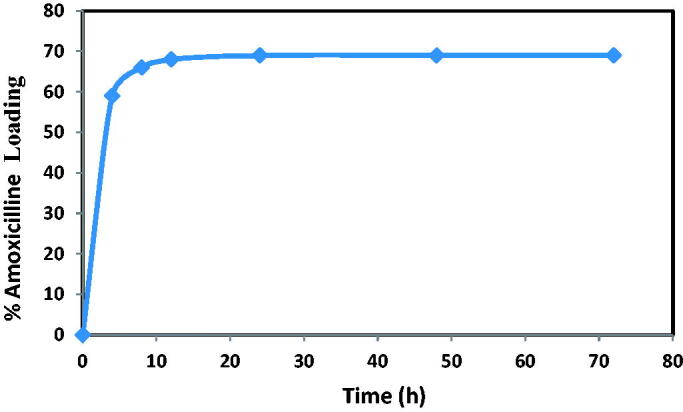
Effect of contact time on amoxicillin loading at initial concentration of amoxicillin 20 mg/L and dosage of NH_2_-SBA-15 = 60 mg.

Figure 12 demonstrates the relation between both the contact time and filled amoxicillin as the desired drug on the bottle (NH_2_-SBA-15). The result illustrates that the charging power is directly proportional to touch before it achieves the equilibrium adsorption. This theoretical study of the process shows that there is a genuinely substantial increase in the drug's load potential during (0–4 hours for amoxicillin) due to the high amount of the developed mesoporous partial surface area. Instead (from 4 to 12 hours for amoxicillin) the drug load with travel time was much less time because this was attributable to a reduction of the available mesoporous material surface area for load, but after 12 hr the high adsorption capacity curve was modified to around horizontal. This indicates that mesoporous particle surfaces are loaded ‘saturated’ or even that equilibrium adsorption was achieved in another phrase. As shown in Figure 12, the best loading time for amoxicillin was 12 hr due to loading between times (12–72 hr) that was only 3% loading up to 12 hr (Manzano et al., 2008).

#### 3.2.2. The initial concentration effect

**Figure 13.**
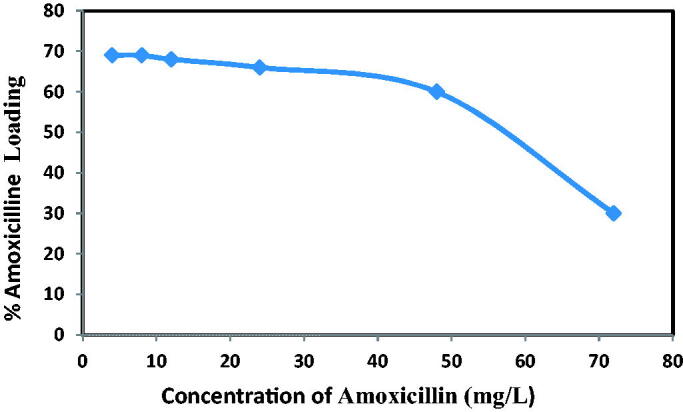
Effect of concentration on amoxicillin loading at contact time 12 h and dosage of NH_2_-SBA-15 = 60 mg.

It examined the effect of the initial amoxicillin concentration on the adsorption action. Figure 13 shows the relation between these 2 factors. When the adsorption reaches the saturated concentration above or at a state of equilibrium, the curve has moved to just about horizontal because there are no more adsorption sites left to fill the drug.

Therefore, the theoretical explanation of the relationship seen between concentration and the loading conditions for the drug ‘amoxicillin’ is originally reverse rationality as the load capacity reduced substantially with an increase in the drug concentration whereas the adsorption also could not exceed the limits (Amount of active sites fill in). Above in, the highest concentration of amoxicillin was 60 mg^−1^ with a maximum load efficiency of 75% (Teodora Tihan et al., 2015).

#### 3.2.3. The dose-effect

**Figure 14.**
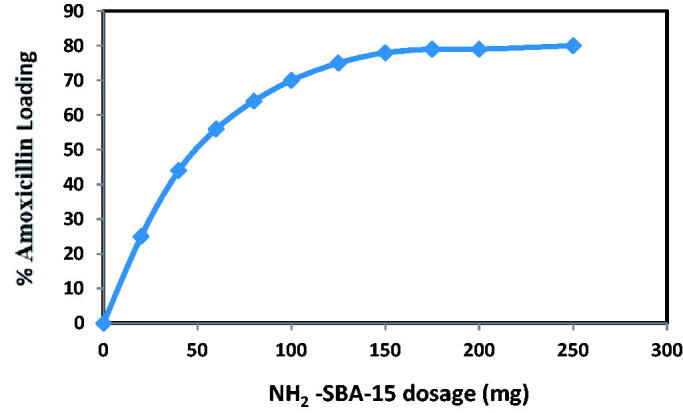
Effect of NH_2_-SBA-15 dosage on amoxicillin loading at initial concentration of amoxicillin 20 mg/L and at contact time 12 h.

The effect of the NH2-SBA-15 dosage on amoxicillin was observed, and the results of this research study were shown in Figure 14. The study was carried out on the effect of the SBA-15 dose-effect after evaluating the contact time and influence of amoxicillin concentration, and after obtaining the optimal values and as per the research investigation. The best concentrations for loaded amoxicillin were 60 mgl^−1^. At twelve hours the concept of the maximum contact time was used. Results in Figure 14 indicate that the dose of NH_2_-SBA-15 increases with the efficacy of the drug charge when it is adsorbed in the equilibrium. The highest dose value can be obtained as this adjustment was introduced and was 60 mg, as the curve shifted to horizontal. It can be attributed to the fact that the change in the medication's load capacity is increased with increase in the adsorbent dose attributed to the rise in adsorption efficiency, which means that more mesoporous practices are required in the mixture, so that more suffuse areas can be used for loading, resulting in a more filled drug. 80% of the best brands of all parameters were produced as per this study (Sevimli and Yılmaz, 2012).

### 3.3. Release of amoxicillin

The UV analysis was performed for 7 hr for the release experiments. For each test, the same quantity of samples considered for the analysis of 1 ml has been transferred to the PBS solution. As a result, increasing concentration of the sample was preserved, and the analysis became even more satisfying.

**Figure 15.**
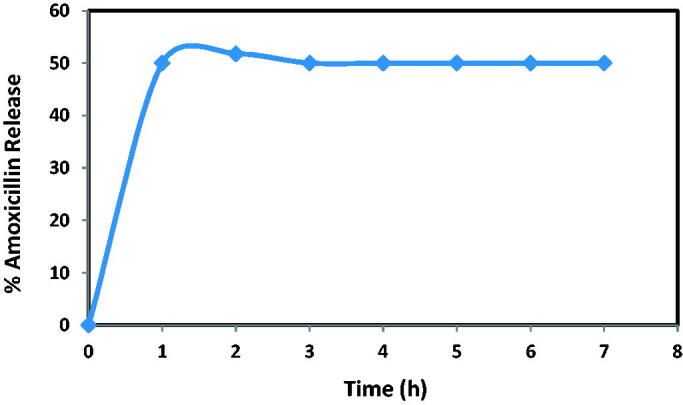
The amoxicillin release profile of NH_2_-SBA-15 sample.

The functional classes of Alkoxysilanes also affected drug delivery, depending on their oscillation. The silica surface has been reported as being hydrophilic with the categories 3-amino propyl. Consequently, the relationships between the mesoporous material and the drugs would cause variations in charging capacity and release rate. As shown in Figure 15, A drug delivery system may also be used to monitor the release of the drugs via SBA-15 particles. The average volume of amoxicillin release (initial burst) is 50.14% and 1 hr within the first hour that could be sustained within the next seven hours. It can be due to (concentration variance) the driving force effect in which the drug shifts from higher concentration from adsorbent to lower concentration and the release increased overtime before the organizations sustained for 1 hour. Functionalized SBA-15 particles are also a good mechanism for regulating drug release (Szewczyk et al., 2019).

It is known that the release property is related to the nature of interactions between the drug and its carrier. A graph of the release percentage versus time for NH_2_-SBA-15 is plotted in Figure 15. As seen from this figure, the release of amoxicillin is decreased for the first time because of the strengthened hydrogen bond between NH_2_ group functionalized NH_2_-SBA-15, and hence a relatively small release of amoxicillin drug was observed. The release percent was gradually increased by decreasing concentration on the surface of NH_2_-SBA-15. Thus, the amoxicillin-loaded NH_2_-SBA-15 was readily protonated, and hence weakened the hydrogen bonding interaction, then further dissociation of amoxicillin molecules occurred leading to an increase in release percent.

### 3.4. Amoxicillin release kinetic model

To define the mechanism that governs the release kinetic cycle, the diffusional equations of ‘Non-Fickian, Korsmeyer–Peppas and Weibull’ models were closely examined (Vora et al., 2013; Ayad et al., 2016):

1. Non-Fickian model and mechanism of diffusional release defined in Equation (3) under n > 1:
  (3) $$\text{Y}={\text{at}}^{\text{n}}$$

When Equation (3) was adopted, the n value would be less than one that did not meet the limits.

2.Model of Korsmeyer–Peppas design had to be less than 0.6 to analyze the release of drugs in the formula given under Mt/M.
  (4) $$\text{Mt/M∞}={\text{kt}}^{\text{n}}$$

When this equation was implemented, the value of Mt/Mt was more than 0.6, which does not meet the design parameters.

4.The Weibull model should analyze the amount of accumulated release from the drug, and it must be equal to or greater than 0.96 in the given formulas under *R*^2^ ‘coefficient of correlation.’
  (5) Ln [ln 1/(1 − f)] = m ln t − ln t

**Figure 16.**
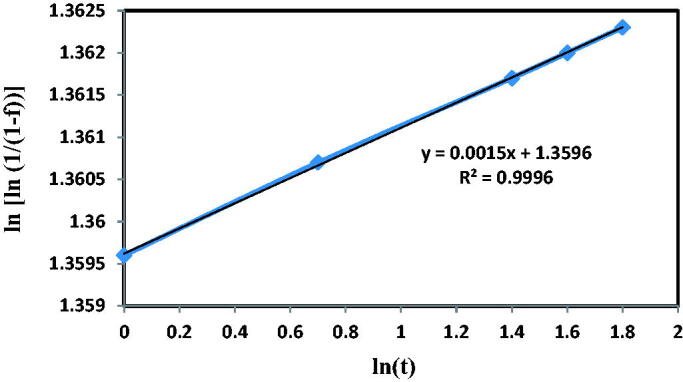
The Release kinetic model of amoxicillin drug delivery system.

Because Equation (5) was applied, the *R*^2^ value was considerably greater than 0.96, which satisfies the boundary conditions, so that the drug release from controlled release systems was simulated effectively as described below.:

Whereas f is the fraction of the total released dose which will be the release of drug ratio at time t. Weibull form parameter represents by M that measures the influence of the drug's unreleased mass ratio on the release rate (0.0015) as seen in Figure 16, while ln t is the intercept (1.3596). Sometimes, t is the released or dissolution time, and the Weibull scale parameter was considered the feature. The meaning of ln [ln 1/(1 − f)] is equivalent to ln t, i.e. the logarithm of t to base e, i.e. plot ln [ln 1/(1 − f)] as opposed to ln t contributing to a linear fashion of m. For the specific situation, Equation (5) has fitted the curves to the sampling data are mentioned in Figure 16; a strong fit of the modulus data was reflected in the determination coefficient (*R*^2^), which is (0.9996) (Manzano et al., 2008; Teodora Tihan et al., 2015).

## 4. Conclusion

Amoxicillin drug can be used as treatment for covid-19 after confirming its efficacy by in vitro assays or clinical trial because already it is widely used and well known even for its dose. The synthesis of the mesoporous silica SBA-15 and NH2-SBA-15 was achieved with a guided post grafting method while a selection of indicators of raw mesoporous material SBA-15 and modified NH_2_-SBA-15 surface was demonstrated by the characterization techniques such as XRD, SEM, FTIR, TGA, and BET surface. Many other dependent variables have been added affecting the performance of the amoxicillin drug maximum load such as initial concentration, contact time, and dosage of NH_2_-SBA-15 resulting in an optimum load capacity of 80% for amoxicillin. With 50%, the release studies for amoxicillin in 1 h were the biggest position.

The diffusion method also contrasted with the released results of drug models of Weibull, and non-Fickian, Korsmeyer-Peppas but the analyzes did not meet the initial conditions-except for the Weibull model. The technique could lead to high load efficiency and a significant decrease in the release of amoxicillin drug making the mesoporous SBA-15 and effective delivery carrier for medicines. We believe that the results of the study may also lead to a wide variety of drug delivery applications in the future.
